# Using Patient Reported Outcomes Measures to Promote Integrated Care

**DOI:** 10.5334/ijic.3961

**Published:** 2018-04-19

**Authors:** Marcel G. M. Olde Rikkert, Philip J. van der Wees, Yvonne Schoon, Gert P. Westert

**Affiliations:** 1 Chair Dept Geriatrics and Radboudumc Alzheimer Center, Radboud University Medical Center, Nijmegen, NL; 2 IQ healthcare Radboud University Medical Center, Nijmegen, NL; 3 Department Geriatrics and Chair Emergency Department, Radboud University Medical Center, Nijmegen, NL; 4 Chair IQ healthcare and Theme leader Health care Improvement Science, Radboud University Medical Center, Nijmegen, NL

**Keywords:** patient reported outcome, PROM, implementation, geriatrics, patient centered care

## Abstract

**Introduction:**

Patient reported outcome measures (PROMs) have been introduced as standardised outcomes, but have not been implemented widely for disease targeted pathways of care, nor for geriatric patients who prefer functional performance and quality of life.

**Discussion:**

We describe innovative multipurpose implementation of PROMs as evidenced by two best practices of PROMs application in geriatric and physiotherapy practice. We show that PROMs can show meaningful outcomes in older subjects’ patient journeys, which can at the same time serve individuals and groups of both patients and professionals.

**Key lesson:**

PROMs can deliver generic outcomes relevant for older patients, may improve patient-physician relationship, quality of care and prediction of future outcomes in geriatric care, if they are valid, reliable and responsive, but still short and simple. A precondition to make the hard tip from research to practice is that PROMs are carefully positioned in the clinical encounters and in electronic health records.

## Introduction

Interest in patient related outcome information is a prerequisite for patient centered care, which has increasingly been recognized as ethical imperative in modern health care. Patient reported outcomes assess aspects of a patient’s health status coming directly from the patient. Such outcomes are assessed with patient-reported outcome measures (PROMs) to quantify reproducible health perceptions of patients, usually through a questionnaire or a single item scale. PROMs can be developed to measure a single dimension of health or a combination of health aspects, collectively known as health-related quality of life. In this paper we aim to systematically describe the various ways in which PROMs can be used in clinical practice, and how integrated care can be served and stimulated by directly taking profit of the information PROMs offer to patients, professionals, health care management, and researchers. However, implementing (PROMs) is not established in current practice of disease oriented pathways of care, nor in global health care for older patients [1]. As we collected best practice data on the application of PROMs in geriatric and physiotherapy practices, we will use these populations as examples, without limiting the lessons learned on integrated care enheancement to these patients.

To meet patients’ preferred outcomes it is necessary to pay more structural attention to global wellbeing outcomes [2]. Structurally applying PROMs may also improve empathy in the patient-physician relationship [3]. However, these various aspects are currently not part of implementation of PROMs in clinical care [4]. Even in the most intensive treatments the structural attention for impact on general wellbeing is limited. While for example the physical domain was meticulously described during trajectories of open heart surgery, notes on subjective wellbeing could only be found in less than half or in one out of ten patients [5]. Therefore, we will subsequently give an update for health care professionals, managers and policy makers based on our long lasting research and clinical practice on the multiple purposes PROMs can serve, and how this can improve their implementation and integration of patients’ and professionals’ interests in daily practice.

## Multipurpose application of PROMs

**Figure 1:**
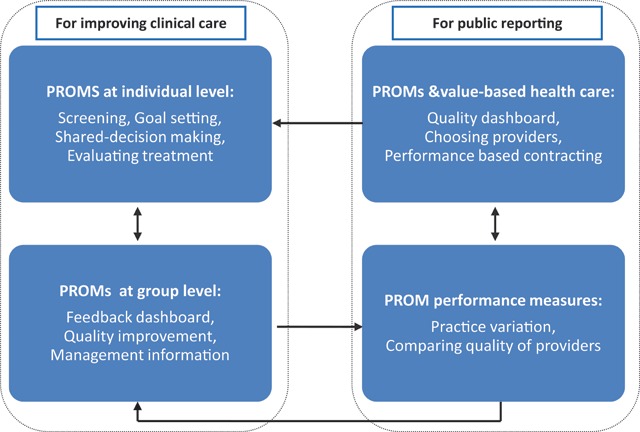
Framework for the innovative multipurpose use of PROMs.

PROMs can play an important role in patient-centered healthcare as discussing health outcomes in the patient-clinician interaction may lead to the patient becoming more involved in goal setting, improve the effectiveness of the patient-clinician relationship, and increase the patient’s self-efficacy [4,5,6]. Especially in frail older patients and other patients with multiple long-term diseases, the use of PROMs may support patients in the process of self-monitoring [7]. PROMs’ feedback to clinicians may increase patient well-being, because it results in patients feeling more comfortable raising and discussing physical, psychosocial and non-medical issues with their clinician during the consultation [7]. If individual PROM-data are aggregated across patients, they can serve as performance measures to compare and improve clinician or organizational quality of care [8]. Thus, PROMs can contribute to add value to health care, but in these early stages of development, also have their drawbacks of limited validity and reliability, privacy issues, and increased administrative burden [8]. The OECD, Organisation for Economic Co-operation and Development (*OECD*), issued a report on the future of health statistics urging for broader PROM use in clinical practice, quality improvement and performance measurement [9]. The Figure 1 summarizes this multi-purpose use of PROMs in clinical care at individual patient level, for internal use by provider organizations in quality improvement, and for external use in performance measurement and public reporting.

## Barriers and facilitators for implementing PROMs

Barriers towards measuring and registering valuable and valid information on wellbeing are plentiful. Health care workers continuously experience time constraints, by which they first try to gather the information needed for an accurate diagnostic work-up, and subsequent therapeutic decision making. Further, finding the medical diagnosis culturally overrules diagnosing what patients really want [10]. Next, in general for administrative purposes, a series of questions have to be asked related to a wide range of quality and safety of care indicators. As we experienced, most older patients understand this continuous hurry for focus on specified information, and hardly dare to bother the medical team with a subjective evaluation of their health. They intuitively prioritize helping the physician localize the objective cause of their complaints with specific complaint-linked answers, instead of discovering what globally matters most to them.

To counteract these barriers, strong incentives and facilitators for serious use of patient reported information are needed. Health care policy makers can importantly facilitate this change towards patient centeredness by emphasizing improvement of the overall quality of care performance and the patient experience in particular, and by making sufficient resources available to support PROMs registration and analyses in the Electronic Health Records (EHR). Including patient reported data plays an important role as part of pay for performance and public reporting systems, but there is no evidence yet what facilitators are most effective. This may also be linked to the personal rewards of working with PROMs, as there is convincing data that it can improve patient-physician communication, and the effectiveness of interventions, especially when patients and professionals take the time and effort to revalue generic information on wellbeing and health related quality of life as health goals and outcomes [8,9,10].

## Predictive value of PROMs

PROMs may not only serve as future assessment targets, but may also have relevant prognostic information. Large studies and subsequent meta-analyses have shown that both in community based populations and in clinical samples, baseline information on subjective health and wellbeing is a relevant predictor of survival [11,12]. High ratings on subjective wellbeing predict 10–25% lower mortality, decline in incidence of ischemic heart disease, and future disability over 7 years in initially healthy cohorts [11]. Therefore, and because wellbeing is an important check for a favorable benefit-harm ratio of the ongoing treatment, general wellbeing and health related quality of life scores are increasingly used as outcome measures for clinical studies in long-term care, oncology, cardiology, and surgery [6,9,11]. These studies showed that subjective health and wellbeing ratings can also be predictive for the intervention-related burden patients can withstand and still adhere to, for example in chemotherapeutic treatment regimens [13]. However, apart from research purposes, generic wellbeing assessment before and after treatment is still rarely used as PROM in most highly specialized medical services [4,10].

## Wellbeing priority of older persons

It is not only the predictive power for survival and the feedback on service quality which makes PROMs important. Older persons often prefer wellbeing over morbidity and survival as outcome measure, especially when they are frail or have a limited life expectancy [3,14,15]. Mostly suffering from multiple chronic diseases these patients prefer a focus on wellbeing, resilience and the positive aspects of life, and consider these more motivating and less stigmatizing than a focus on survival, frailty, and disease related outcomes [16,17]. Likewise, symptomatic relief from a palliative care perspective may often fit better than cure directed treatment registration, with a focus on objective disease activity.

Such PROM questions that professionals can use to ask for wellbeing and self-rated health can be divided in objective and subjective questions corresponding to the three different health domains relevant in the biopsychosocial paradigm (Table 1).

**Table 1:** Examples of objective and subjective PROM questions corresponding to physical, mental and social health domains.

	Objective question	Subjective question

**Physical domain**	How long did you perform activities requiring some physical effort yesterday (e.g. walking, shopping, hobbies, etc.)?	How would you rate your physical health today?
**Mental domain**	How long did you feel in a good mood yesterday?	How would you rate your mental health today?
**Social domain**	How long did you perform social activities yesterday (e.g. family contacts, meeting friends or neighbors, phone calls, card games, etc.)?	How satisfied are you with your social activities today?

When aiming for assessment of wellbeing by a PROM, there are numerous validated “health-related quality of life” (HRQOL) questionnaires that address relevant domains systematically, both generic (e.g. SF-36, EQ-5D), and disease specific (e.g. in dementia: QoL-AD, DQI). Using a valid PROM to assess wellbeing really at the beginning of a clinical encounter however may hinder an open dialogue on these fundamental issues, and a compassionate start of the patient-physician relationship. Therefore, clinicians still need to start with open questions such as “How are you today?” followed up by questions such as: “What matters to you most at this stage of life?” Moreover, we need to give the patient and ourselves time to reflect on this. To harmonize and be able to compare wellbeing and subjective health outcomes within and between patients, we can follow up with one of the abovementioned instruments or ask a single standardized question. This question may ask for rating global health from 0 (worst possible) to 100 (best possible health), or for whether they currently enjoy life with categorical answer categories (from “never” to “always”) [11,12,16,17]. These single questions have proven to be valid and reliable in large studies, and can simply be specified to the physical, psychological, and social wellbeing domains. Smart EHRs could pick up such information easily, even by voice recording. By limiting the answer to the current situation, PROMs can be monitored repeatedly, and as they are independent of intact memory, the questions can also be used in mild stage dementia. In sum, revaluing and focusing on PROMs from the start of our clinical encounters thus harbors multiple opportunities to add value to current medicine, not the least because all patients highly value a genuine interest in their wellbeing. This may even lead to the disruptive rearrangement of reimbursement for clinical services based on PROMs, which might be the strongest incentive for value based health care [18].

## Conclusions from best practices

We conclude with two evidence-based examples from different settings, which illustrate how clinicians and patients may benefit when PROM registration is implemented in regular practice. One example is based on geriatric care and the other on physiotherapy care in the Netherlands. As a result of these best practice experiences the use of PROMs for clinical practice and quality improvement now is becoming integrated care in Dutch geriatrics. The examples are different from the numerous studies that describe development and validation of PROMs, as in both cases they were brought beyond the tipping point that separates the educational setting and health services research from clinical practice. These best practices show that also single patients can have direct benefit as their PROM rating is directly presented to the clinician, who can adapt the management plan accordingly (**Box 1**). The physiotherapy case shows that large scale application of these PROMs can be reached by having access to an EHR that can generate feedback presentation of aggregated results (**Box 2**).

**BOX 1: Older patients’ PROMs**

Mrs. A, was admitted to a post-acute geriatric ward being injured from het most recent fall, also suffering from depression, hypothyroidism, cerebrovascular disease, and severe hip osteoarthritis, but still living by herself. What matters for patients like Mrs. A, is poorly reflected in disease directed outcome measures, which do not focus on general wellbeing. Therefore, the Older Persons and Informal Caregivers Survey Minimum Data Set (TOPICS-MDS) was developed and validated as a PROM, and between 2010 and 2013 applied in more than 40,000 older persons and 4,000 informal caregivers [20]. The TOPICS-MDS contains 42-items and was used successfully nationwide to evaluate effectiveness of more than 60 health care innovations as part of the Dutch National Care for the Elderly Programme.

The Dutch Geriatrics Society recently decided to develop, validate and routinely use an 18 item short form TOPICS-SF as PROM for their patients. This short version still covers subjective health, quality of life, capability for activities of daily living, pain, and psychological wellbeing, with a composite endpoint validated against preferences of community dwelling older persons [21]. For example, 223 patients subsequently admitted to geriatric wards in the first three Dutch hospitals implementing these PROMs, were interviewed on admission and at discharge, and by telephone one month after discharge, and 115 patients (mean age 82.8 ± 7.3 years, 57.8% female) participated. This 52% compliance to PROM reporting is high, taking into the account that all patients were highly frail and only 27% had no cognitive decline. In this routine care group 15.6% reported no problems with self-care on admission, which increased via 16.3% at discharge to 30.3% after one month. Moderately well or good subjective health reporting increased from 69% on admission to 90% one month after discharge. These results were shared with professionals, and directed attention to the profile of patients without improved wellbeing.

The clinical management plan for Mrs. A was targeted to goals she preferred in the PROM data on admission, and her wellbeing improved as checked by her discharge-PROM: she had less pain, was more able to (un)dress herself, and showed increased self perceived health. This reassured the clinician in the timing of discharge.

**BOX 2: PROMs in physical therapy**

Mr. B is a patient with Chronic Obstructive Pulmonary Disease (COPD) and was enrolled in a pulmonary rehabilitation programme after several exacerbations. His physical capacity is severely limited. Prior to therapy he completed the Clinical COPD Questionnaire which contains 10-items asking about symptoms, functional limitations and psychosocial dysfunction.

Measuring wellbeing of patients like Mr. B., including physical and social functioning, is nowadays common practice in Dutch physical therapist care. The goal of physical therapist care is aimed at improving (physical) activities and participation of patients in daily life. To guide goal setting And shared decision making, physical therapists use PROMs such as the Clinical COPD Questionnaire to identify relevant symptoms and limitations in social activities. However, until recently the data collected were not being used for evaluating the quality of care and for guiding quality improvement. Thus, in 2013 the Royal Dutch Society for Physical Therapy launched the program “Quality in Motion”. The aims of the program are to support goal setting, monitor outcomes, give feedback on quality of care, and prove added value to payers and patients [22,23].

Via a web-portal, physical therapists can real-time compare their age, sex and therapy stratified treatment results with their colleagues and with a national benchmark. Peer assessment strategies are also available as effective method for quality improvement cycles, in which physiotherapists assess each other’s performance based on feedback reports generated with data in the registry [23,24]. Professionals are effectively being evaluated by online self-assessment and by their peers based on PROMs, and provide each other with performance feedback that triggers reflection on outcomes, which all have shown to result in overall quality of care improvement [25,26].

After the rehabilitation programme Mr. B was able to cycle for an hour and join his friends again for their weekly bowling event. Mr B’s score on the Clinical COPD Questionnaire has improved substantially. His scores are added to the database used by his physical therapist to compare outcomes with other colleagues in the regional network of specialised physical therapists.

Before implementation of PROMs in clinical practice, feasibility of patient burden, logistics of workflow impact, display of results, and administration frequency should be carefully evaluated, just as psychometric properties and applicability of the outcomes [15]. Professionals, both experienced and just entering health care practice should be adequately trained in adequate acquisition of patient related information (Table 2). The bottom line of the best practices presented however is that it is possible to routinely implement PROMs, even along the majority of the complex geriatric patients in such a way that it makes a difference to patients and to service provision [19]. In sum, yes we can assess what matters to patients during their patient journey, and yes we should do it.

**Table 2:** Key points in starting routine implementation of Patient Reported Outcome Measures (PROMs).

Key points in multipurpose application of PROMs
Standardize outcome measurement by selecting and routinely using patient reported outcome measures (PROMs) that are valid, reliable and responsive, but still short and simple. Implement PROMs in the clinical encounters and electronic health records. Learn to apply open and closed, objective and subjective PROM questions on wellbeing, especially for patients with multiple chronic diseases. Repeat PROMs during the patient journey, as this can improve patient-physician relationships, guide burdensome clinical treatment, and assist in treatment adherence prediction. Systematically improve your practice by individual and peer group feedback on the PROMs. Secure organizational commitment, practical and educational support in the PROM implementation process. Clarify the incentives for all professionals involved, but emphasize intrinsic rewarding of improved patient-physician relationship and improved professional satisfaction.

## Author Informations

Dr. Philip Van der Wees is a physical therapist, senior researcher and principal investigator at the Scientific Institute for Quality of Healthcare (IQ healthcare) of Radboud university medical center. In 2017 he started a part time position at the School of Medicine and Health Sciences at the George Washington University in Washington, D.C. The research and teaching activities of Philip van der Wees are aimed at implementation science, quality improvement, and evaluation of healthcare quality. Central focus of his research is assessing health care outcomes, specifically patient-reported outcomes.

Dr Schoon is geriatrician and head of the Department of Emergency Medicine of Radboud university medical center. She is Principal Clinician and innovated quality of care on for example transcatheter aortic valve inplants, orofacial oncology, shared decision making and other aspects related to quality of care for older patients.

Dr. Gert Westert is head of IQ healthcare and professor of healthcare research/quality of care. He studies the relation between quality and costs in healthcare.

Dr Marcel Olde Rikkert, is head of the department of Geriatric Medicine at the Radboud university medical center. He is principal investigator on quality of care for frail older patients, specifically related to dementia and complex interventions.
